# Life Cycle Reversal in *Aurelia* sp.1 (Cnidaria, Scyphozoa)

**DOI:** 10.1371/journal.pone.0145314

**Published:** 2015-12-21

**Authors:** Jinru He, Lianming Zheng, Wenjing Zhang, Yuanshao Lin

**Affiliations:** 1 Marine Biodiversity and Global Change Research Center (MBiGC), Xiamen University, Xiamen, China; 2 Fujian Provincial Key Laboratory for Coastal Ecology and Environmental Studies (CEES), Xiamen University, Xiamen, China; 3 College of Ocean and Earth Sciences, Xiamen University, Xiamen, China; 4 Zhejiang Provincial Zhoushan Marine Ecological Environmental Monitoring Station, Zhoushan, China; UC Irvine, UNITED STATES

## Abstract

The genus *Aurelia* is one of the major contributors to jellyfish blooms in coastal waters, possibly due in part to hydroclimatic and anthropogenic causes, as well as their highly adaptive reproductive traits. Despite the wide plasticity of cnidarian life cycles, especially those recognized in certain Hydroza species, the known modifications of *Aurelia* life history were mostly restricted to its polyp stage. In this study, we document the formation of polyps directly from the ectoderm of degenerating juvenile medusae, cell masses from medusa tissue fragments, and subumbrella of living medusae. This is the first evidence for back-transformation of sexually mature medusae into polyps in *Aurelia* sp.1. The resulting reconstruction of the schematic life cycle of *Aurelia* reveals the underestimated potential of life cycle reversal in scyphozoan medusae, with possible implications for biological and ecological studies.

## Introduction

Pelagic cnidarians have received significant attention as important competitors and predators in marine ecosystems [1–4]. While the hypothesis that global jellyfish populations have increased over the last few decades is still under debate [5–8], their ecological and socio-economic impacts [9–11], causes and drivers for blooming [12–14], and biological implications [15, 16] are well-studied.

As one of the major contributors to jellyfish blooms, moon jellies (*Aurelia* spp.) are one of the most thoroughly studied scyphozoans [17, 18]. The cosmopolitan genus *Aurelia* was long considered to occupy neritic waters between 70°N and 40°S, with few valid species identified largely due to ambiguous morphological characteristics [19]. However, genetic analyses suggest that at least 16 sibling species exist [20, 21]. While most *Aurelia* species are reported to have restricted geographic ranges, *Aurelia* sp.1 is considered a successful invasive species globally distributed across major warm-temperate regions, with a possible origin from the Northwest Pacific [21–23].

Apart from anthropogenic and environmental causes [18, 24–26], life cycle flexibility also contributes to *Aurelia* blooms [15, 27]. The typical life cycle of *Aurelia* comprises the following transitions: benthic polyps asexually produce free-swimming ephyrae, which develop into the medusae, and medusae produce sperms and eggs that fertilize to form planula larvae which develop into polyps [28]. While medusae are capable of long distance dispersal, the polyps also possess great potential in population amplification by various means of asexual reproduction through which novel structures (e.g. podocysts [29] and free-swimming propagules [30]) are produced that help overcome unfavorable environments.

Modifications of typical life cycles are not rare in the Cnidaria, though life history characteristics are still important diagnostic features for many taxonomic groups in this phylum [31–33]. Diverse types of asexual reproduction and encystment are found in Anthozoa [34, 35], Cubozoa [36, 37], and Staurozoa [28, 38], and life cycles in Hydrozoa are characterized by an unparalleled plasticity [39]. The first case of reverse development in Cnidaria was discovered in the scyphozoan *Chrysaora hysoscella*, which is capable of back-transformation from ephyrae to polyps in unfavorable environmental conditions [40]. Many other scyphozoan ephyrae, including *Rhizostoma pulmo* and *A*. *aurita*, also undergo ontogeny reversal, first regressing into planuloid masses of cells, and then growing into polyps over weeks or months [41, 42]. Despite the wide plasticity of scyphozoan life cycle, reverse development has not been documented in sexually mature medusae, which is in contrast to the unparalleled potential of some hydrozoan species [43–46]. In this study, development patterns of *Aurelia* sp.1 were followed, and life cycle reversal processes of both juvenile and sexually mature medusae were recorded, which represents the first time such phenomena have been documented in a scyphozoan.

## Materials and Methods

### Field sampling and laboratory culturing

**Fig 1:**
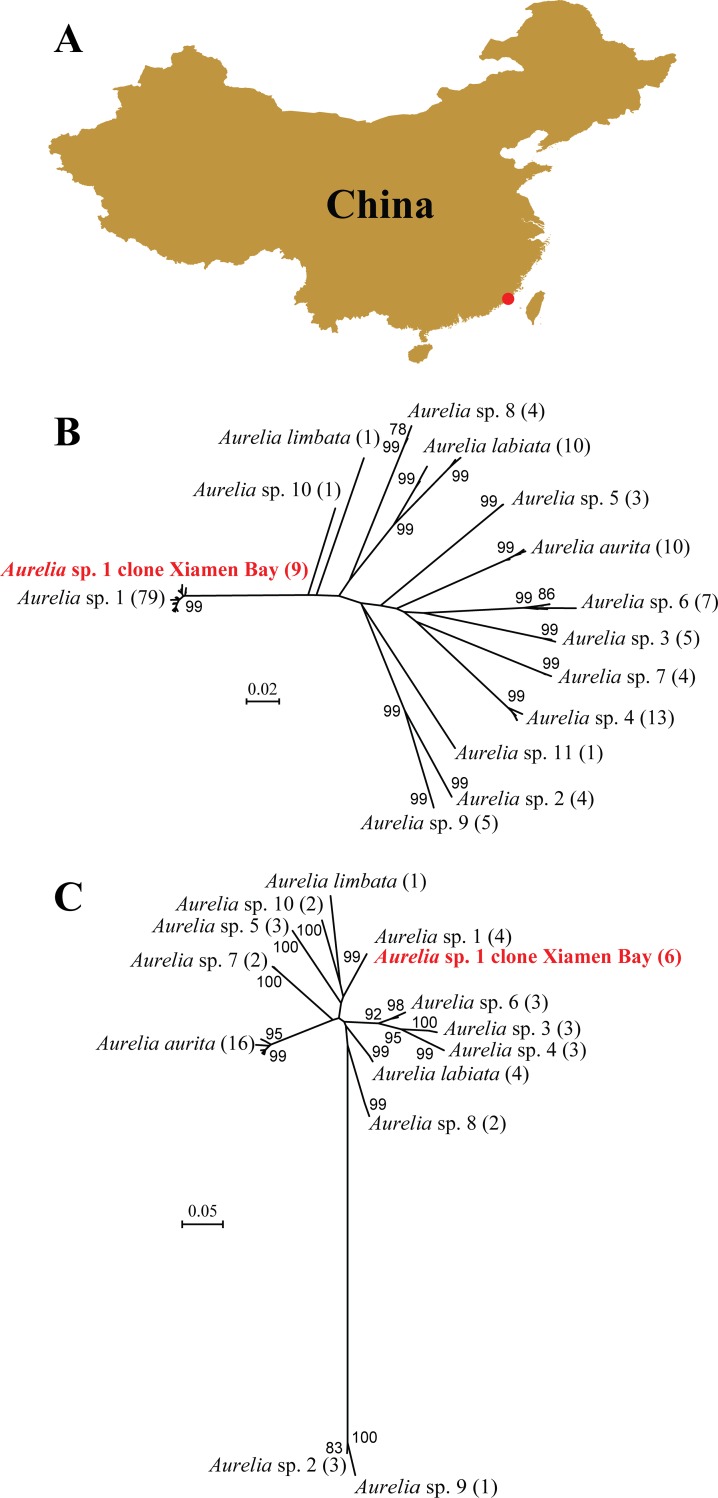
Species information of *Aurelia* sp.1 in Xiamen Bay, East China Sea. A: sampling location for *Aurelia* sp.1 from Xiamen Bay, which was indicated as the red dot in the map. B and C: Neighbor-Joining cladogram of *Aurelia* based on mitochondrial COI (B) and nuclear ITS (C) sequences, bootstrap values higher than 70 were shown close to each branch node, number of sequences belonging to the same species were indicated in the bracket following the species name, and sequences of *Aurelia* sp.1 obtained in this study were highlighted.

Two ephyrae were collected from Xiamen Bay (24.4514°N, 118.0753°E), East China Sea (Fig 1A), using a plankton net (mesh size: 505 μm) on April 17, 2011. They were then brought back and reared to adult medusae in a 15 × 15 cm glass tank with filtered sea water (filter mesh size: 50 μm). Both animals were male, with sperm release recorded in the first individual on October 14^th^ and the second in late November, 2011. The latter animal was found damaged due to collision with a plastic tubing for air flow after an overnight aeration, and then was preserved using 95% ethanol in January, 2012. The remaining medusa sank onto the bottom of the tank and was no longer able to sustain swimming by early September, 2012. The tissue fragments of this specimen were collected and transferred to a new tank with freshly filtered sea water. A single polyp with 3 developing tentacles was first noticed by chance on top of the degraded fragments on November 23, 2012, followed by several polyps across the degenerating fragments in the following days. These polyps were collected and transferred to a new tank. The first ephyra developed from these polyps on January 10, 2013. The liberated ephyrae and derived medusae were used as materials for this study.

### Morphological and molecular analysis

All animals were fed with *Artemia* sp. nauplii daily, with leftover nauplii removed and water changed every other day after feeding. Air bubbles were pumped into the upper column of the aquarium by a micro pump for medusae (larger than 5 cm in diameter) and polyps (over 10 ind·cm^-3^ on average). Ephyrae and medusae intended for life cycle experiments were fed three times a day, and kept in a hypoxic environment without changing water or aeration (over 300 individuals for ephyrae, 100 for juvenile medusae, and 20 for adult medusae in each tank, respectively). Once individuals settled to the bottom of the aquarium, they were then gently transferred to another new tank, where they were no longer fed. Water temperature was kept at 22±5°C and salinity at 31±4 ppt. Morphological changes were recorded with a Zeiss SteREO Discovery V12 and Olympus BX51 microscope.

For species diagnoses, partial mitochondrial COI (primer: HCO2198-taaacttcagggtgaccaaaaaatca, LCO1490-gtcaacaaatcataaagatattgg) [47] and nuclear ITS (primer: jfITS1-5f-ggtttccgtaggtgaacctgcggaaggatc, jfITS1-3r-cgcacgagccgagtgatccaccttagaag) [20] gene fragments were analyzed from both medusae and polyps according to reference [48]. The COI and ITS sequences were aligned using ClustalX V2.1 [49] with additional *Aurelia* entries from GenBank. Genetic distance was determined by MEGA 6.06 [50] with Kimura-2-Parameter model, and Neighbor-Joining phylogenetic analyses were performed using MEGA with bootstrap values calculated from 1000 replicates, respectively. Genbank accession numbers of *Aurelia* sp.1 from Xiamen Bay were KF962060-KF962065 and KJ733900-KJ733902 for COI, and KF962383-KF962388 for ITS, with additional KF962395-KF962400 and KJ733908-KJ733912 for mitochondrial 16S (primer: 16SH-cataattcaacatcgagg, 16SL-gactgtttaccaaaaacata) [51], respectively. Taxa employed in this study and their GenBank accession numbers are listed in S1 Table.

### Ethics statement

All data included in this study were collected using non-destructive sampling methods. No specific permissions were required for the locations or activities of our field studies since it is a public port base, no endangered or protected species were involved either.

## Results

### Species diagnosis

The observed maximum intra-specific genetic distance in the genus *Aurelia* was 0.092 for COI and 0.035 for ITS, while the minimum inter-specific genetic distance was 0.120 for COI and 0.054 for ITS, respectively. The K2P genetic distance between individuals from Xiamen Bay and those from other locations of *Aurelia* sp.1 ranged 0.003–0.018 for COI and 0 for ITS. Thus barcoding gap [52] was observed for both genes, and individuals collected in this study were matched to *Aurelia* sp.1. Phylogenetic analyses based on COI (Fig 1B) and ITS (Fig 1C) sequences also supported that our specimens formed monophyletic clade with *Aurelia* sp.1 sequences from GenBank, and separated from all the other known *Aurelia* species, which were consistent with published data and predicted geographic ranges [21].

### Direct polyp formation from degenerating juvenile medusae

**Fig 2:**
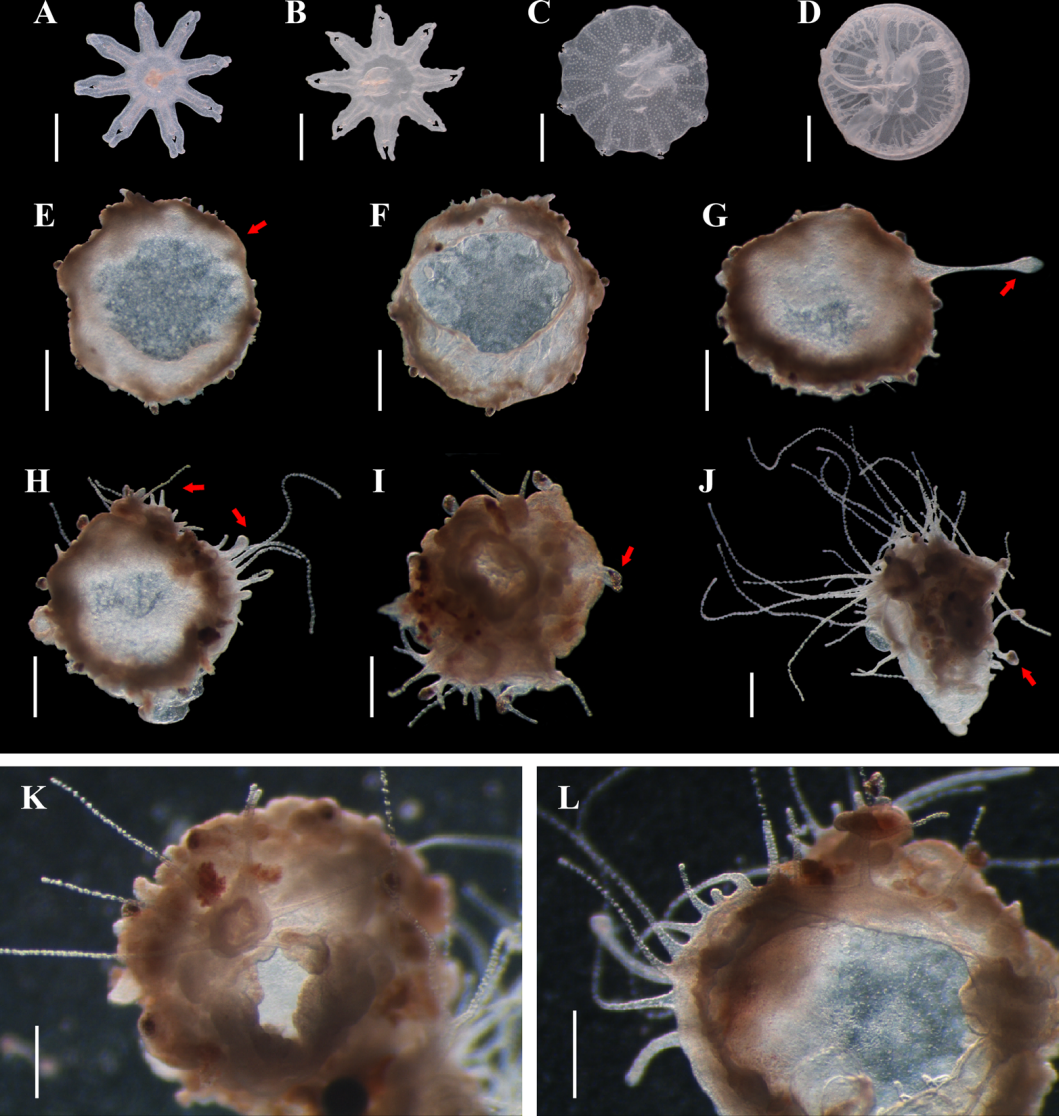
Direct polyp formation from degenerating juvenile *Aurelia* sp.1 medusae. A-D: normal development from ephyrae to juvenile medusae, showing individuals of newly released (A), 5-day (B), 10-day (C) and 20-day (D) old, respectively. E, F: aboral (E) and oral (F) view of a 25-day old medusa after 5 days post settlement. G-L: juvenile medusae during reverse transformation. Scale bars = 0.2 mm (A, B); 0.5 mm (C-L). Arrows showed degeneration of medusa tentacles (E), occurrence of polyp stolon (G), development of polyp tentacles (H), and remains of medusa rhopalia (I and J).

Under normal conditions (i.e. with sufficient food supply and water replacement), the ephyrae would develop into juvenile medusae in about 10 days post liberation, and then reach maturity in the following months at around 18°C (Fig 2A–2D). However, individuals of about 10–25 days old would often aggregate on the bottom of the tank when the aquarium became overcrowded. Those settled to the bottom first showed reduction in the general structure of the body (‘degrowth’ and ‘morphoretrogression’ according to [53]), and umbrella pulsation ceased within two days. Their oral arms and tentacles were then resorbed, with inner structures between the two exumbrella layers gradually fusing together and then disappearing in about 24–36 hours (Fig 2E and 2F). New polyp tentacles emerged from all over the subumbrella surface area, but mostly along the umbrella margin where the original medusae marginal tentacles situated, with (Fig 2G) or without (Fig 2H and 2I) the occurrence of a stolon. Finally, a new polyp mouth developed at the central position among each cluster of newly derived tentacles, with polyp colonies being established in the following weeks (Fig 2J–2L). Certain structures, like the manubrium of young medusae (Fig 2I and 2K) and rhopalia of most individuals (Fig 2E and 2L) remained morphologically unchanged during the early stages, but were lost when the polyp colonies eventually took over. The duration of the whole transformation process was about 5–7 days from the settlement of free-swimming juvenile medusae to newly formed polyps with functional mouths. The process would be postponed when individuals undergoing reorganization (Fig 2E and 2F) were disturbed, but once new tentacles or stolon emerged, it would be accelerated. The reverse transformation was quite common when large quantities of medusae were cultured together with limited space and air supply, showing a relatively high success rate (134 colonies from 150 medusae).

### Direct polyp formation from medusae tissue fragments

**Fig 3:**
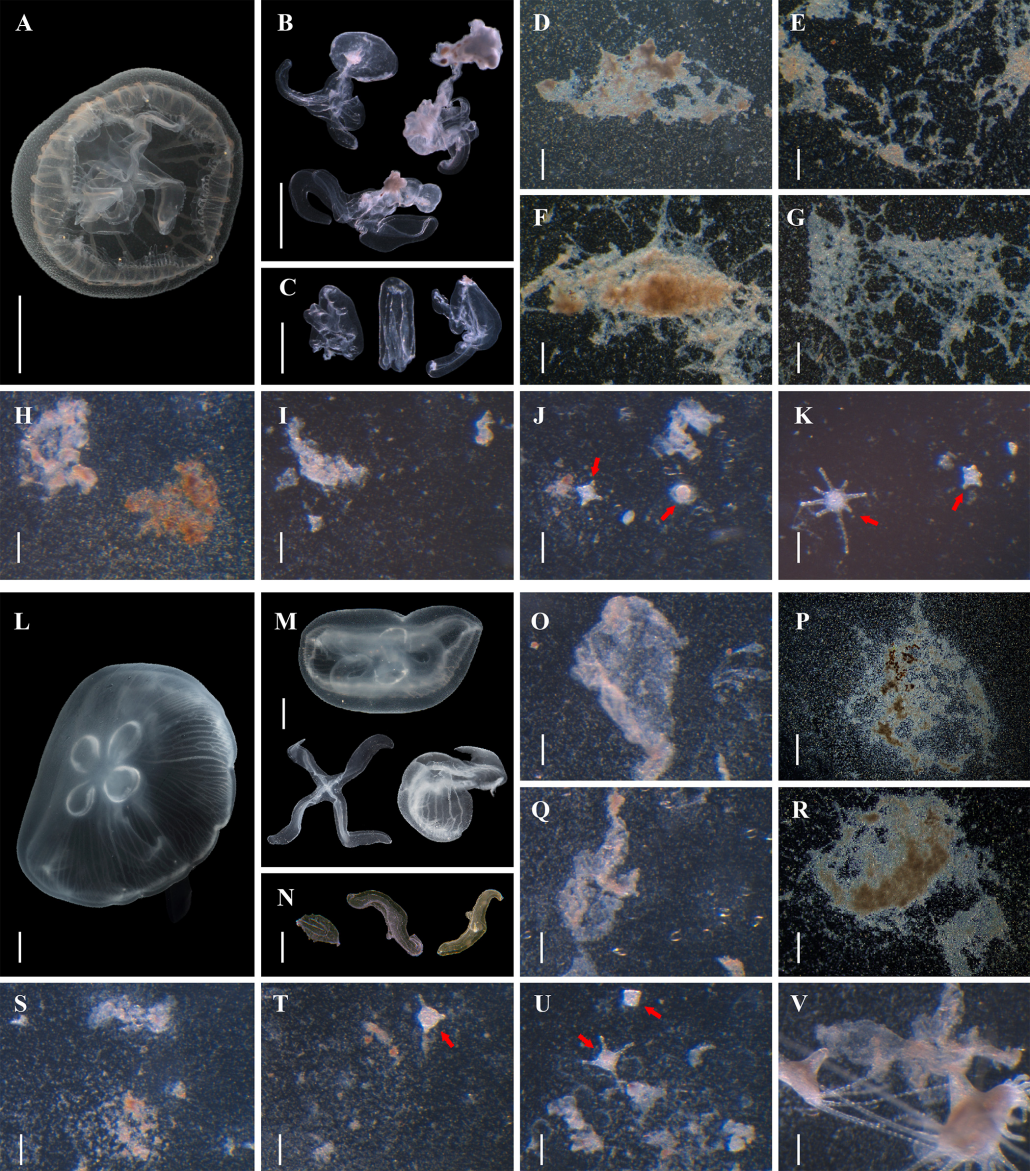
Direct polyp formation from *Aurelia* sp.1 medusae tissue fragments. A: a 25-day old medusa. B: juvenile medusae 5 days after settlement. C: oral arms 1 month after settlement. D-G: tissue fragments from juvenile medusae. H-K: polyps arose from juvenile medusae fragments. L: a 1-year old male medusa (collected from the field). M: settled broken or fragmented adult medusae. N: oral arms 3 months after settlement. O-R: tissue fragments from adult medusae. S-V: polyps arose from adult medusae fragments. Scale bars = 1 mm (A, B, C); 0.2 mm (D-G); 0.1 mm (H-K, O-V); 1 cm (L-N). Arrows showed newly developed polyps.

The whole organisms or fragments from both young (Fig 3A) and mature (Fig 3L) medusae were capable of undergoing life cycle reversal in a manner more comparable to published descriptions in other cnidarians [43–46]. Medusae under starvation or physical stress (e.g. hypoxia or mechanical injury) settled to the bottom of the aquarium and shank in size (degrowth). These individuals gradually ceased pulsation and contraction, which was followed by umbrella degradation (Fig 3B) or fragmentation (Fig 3M). The disassociated tissue attached to the substrate proceeded to conduct reconstruction (Fig 3D–3I and 3O–3S), as rudiments developed, tentacles arose and mouths formed (Fig 3J, 3K and 3T–3V), leading to colony formation. Interestingly, the oral arms or their fragments were able to maintain muscular contraction, spread over the contacting surface, and even envelope prey months after detachment (Fig 3C and 3N). The reconstruction stage (morphoretrogression) lasted from days to months, while the whole process of reverse development from tissue degradation to polyp formation ranged from seven days to two months. A shorter duration of less than 2 weeks was observed for two 30-day old medusae in late spring (T = 18±5°C), compared to 2 months for a 1-year old male individual (Fig 3L) in winter (T = 12±2°C). Although nearly all the degenerating medusae or their pieces left degraded fragments onto the substrates, the success rate and duration of transformation varied as both young and adult individuals (over 5 cm in diameter) showed different values in different seasons.

### Direct polyp formation from living medusae

**Fig 4:**
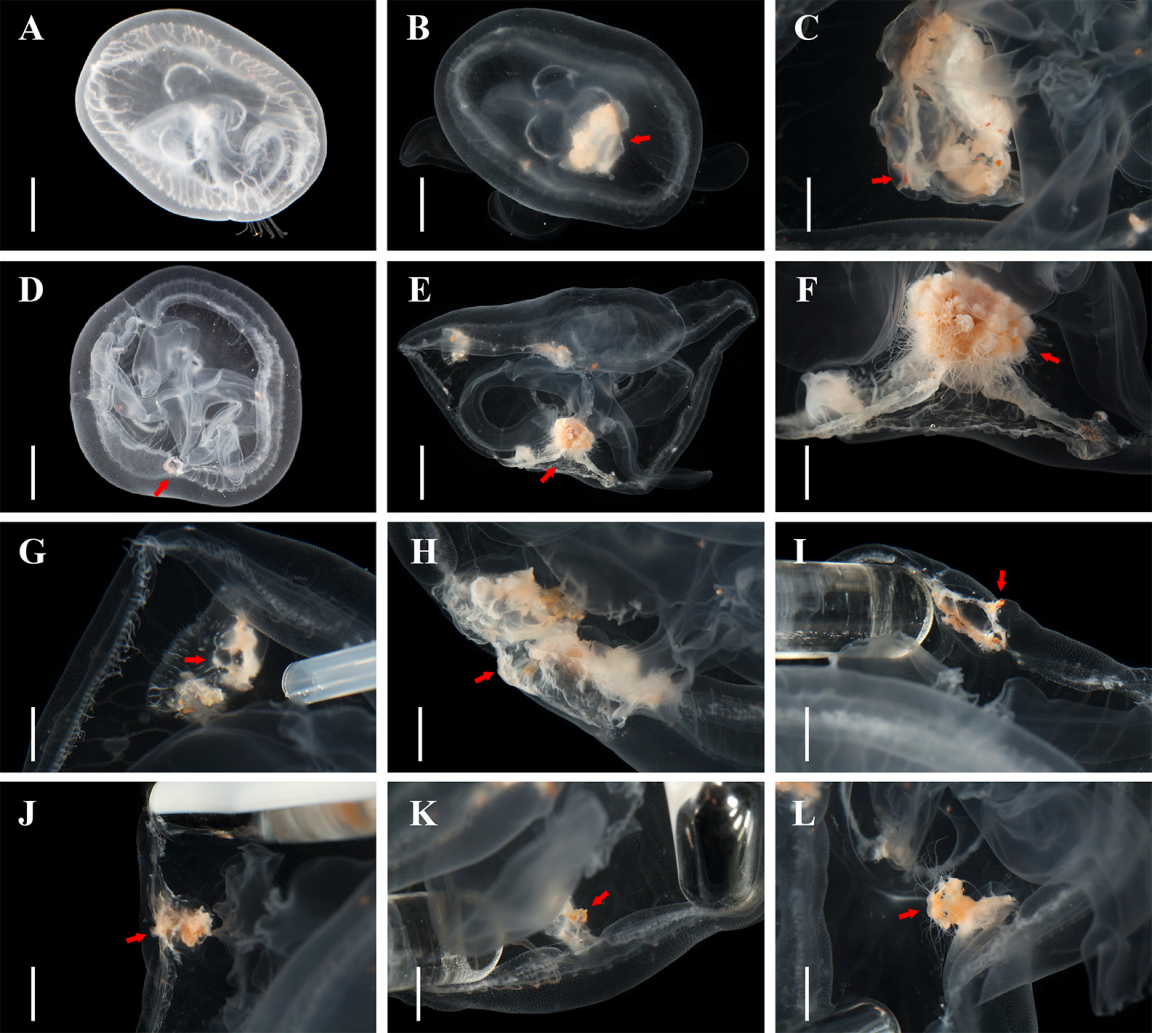
Direct polyp formation from living *Aurelia* sp.1 medusae. A, D: 3-month old medusae. B, C: the same individual as (A) after 75 days, showing the thickened layers and projecting pointed ends at different scales. E-G: the same individual as (D) after 75 days, showing the derived colonies (E and F) and thickened layers (G) at different scales. H-L: proportions of living medusae at different stages of direct polyp formation. Scale bars = 1 cm (A, B, D, E); 5 mm (C, F, G-L). Arrows showed transformation process at different stages.

Three-month old medusae reached about 5 cm in diameter (Fig 4A and 4D) in the laboratory before sexual maturity was noticed. In normal individuals, prey captured by marginal tentacles or attached to the exumbrella are transferred by the four oral arms to the gastovascular cavity (stomach), where it is digested and distributed through the branched gastric canal system. However both the marginal food pouches and stomach pouches, and the branch nodes of the canal system as well, could accumulate prey particles when medusae were frequently overfed, and then further expand to some extent later. Interestingly, repeated physical injury at the same position, e.g. umbrella punctures induced by air bubbles swallowed or pointed pipelines, would also cause such pouch expansions. The ectoderm of these expansions gradually thickened and became less transparent (Fig 4B–4D and 4G–4I), and rudiments (stalk and calyx) appeared upon the pointed ends projecting from the thickened layers (Fig 4J and 4K). Polyp tentacles arose from these rudiments with mouth openings (Fig 4L), and finally colonies were established (Fig 4E and 4F). These colonies developed rapidly, and eventually fell off the free-swimming medusae by umbrella pulsation, after which the polyps settled onto the substrates. The medusae umbrella became no longer round in shape (Fig 4E) and they swam more slowly as these marginal polyp colonies grew, but would recover after the polyps detached. The duration of ectodermal thickening and colony development varied widely. One individual (Fig 4D) we followed in the laboratory took around two months to develop its first polyp following our first observation of the thickened ectoderm, and another one and a half months until the colony detached. The rate of successful colony formation in the laboratory was not high: only three individuals developed polyps out of the 22 medusae reared to the adult stage (over 5 cm in diameter), even though nearly two-third of the medusae developed the thickened subumbrella portions as they grew older.

## Discussion

Life cycle modification in scyphozoans has been significantly underestimated, owing to the limited cases reported. Although back transformation was first described in this class, life cycle reversal in scyphozoan has only been observed in the ephyra stage [42]. The present study describes the unprecedented potential of life cycle reversal in *Aurelia* sp.1 by showing that the polyp stage can be achieved directly from both juvenile and sexually mature medusae. And the derived polyps in all cases also retain the ability of strobilation and asexual reproduction in our observations.

**Fig 5:**
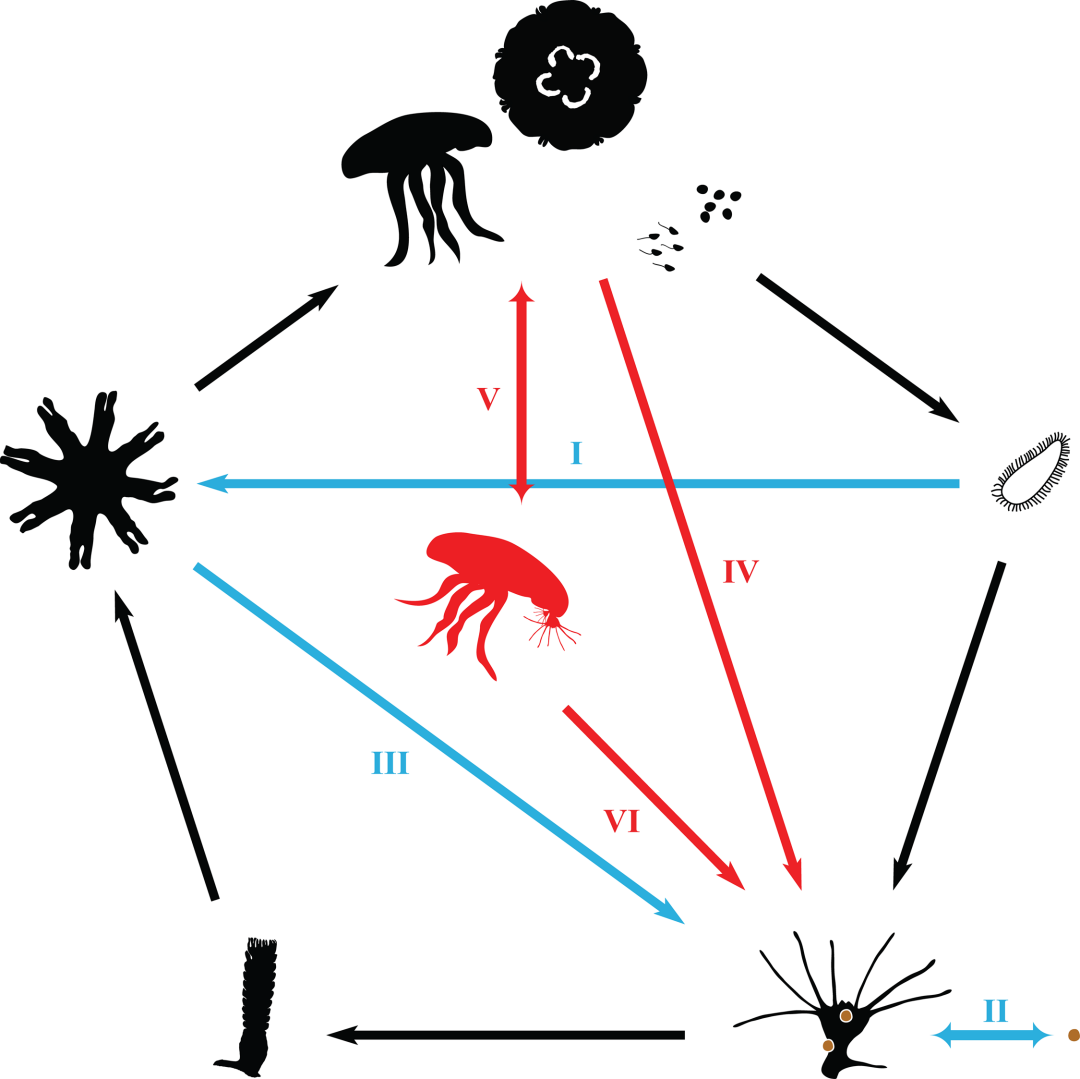
Schematic life cycle of *Aurelia*. Normal development traits and stages were presented with black illustrates and arrows, whereas modifications of the typical life cycle were drawn with colored objects (Red: process described in this study; others: process published with references herein). I: direct development of planula; II: production of elongated stolons, podocysts, and free-swimming propagules from scyphistoma; III: reverse development of ephyra; IV: direct polyp formation from degenerating juvenile medusa and medusa tissue fragments; V: direct polyp formation from living medusa; VI: polyp colony release from medusa-polyp complex.

In light of these observations, a revised life cycle is illustrated in Fig 5. The canonical life cycle of *Aurelia* sequentially includes a fertilized egg, planula, scyphistoma, strobila, ephyra and medusa [28]. However, the planula may undergo vegetative multiplication or develop directly into an ephyra shortly after settling (Fig 5I), without the formation of a scyphistoma [54]. The scyphistomae usually reproduce asexually by formation of buds similar in form to the parent polyp or by longitudinal fission [55], but may also produce elongated stolons, podocysts [29, 56], and free-swimming propagules that settle and develop into new polyps (Fig 5II) [30]. The ephyra (Fig 5III), as well as the juvenile and sexually mature medusa (Fig 5IV) may undergo life cycle reversal in which polyps are formed directly from whole individuals or just proportions of their fragments under certain circumstances. Moreover, the free-swimming medusa may also directly give rise to polyps and become a medusa-polyp complex (Fig 5V), and the complex will release the polyp colonies (Fig 5VI) and turn back into free swimming medusa again.

Beyond the growing interest in addressing life history modifications, the molecular and cellular basis supporting the various processes of life cycle in either the genus *Aurelia* or the phylum Cnidaria are still far from clear. In comparison, the medusa-polyp complex of *Aurelia* sp.1 resemble those in several Hydrozoa species (e.g. *Clytia mccradyi*) that are capable of asexually budding polyps directly on the body of the medusa, either on the manubrium or on the radial canals [57–59]. While the direct polyp formation from both degenerating juvenile medusa and medusa tissue fragments of *Aurelia* are both comparable to reverse development observed in two hydrozoans, *Turritopsis dohrnii* [43, 44, 46, 60, 61] and *Laodicea undulata* [45, 62], based on the common regressing stage and the extent of transformation potential. Both the blastostyle budding in *C*. *mccradyi* and the reverse development in *T*. *dohrnii* require not only the ectoderm interstitial cells, but also the endodermal lining of the canal system that give rise to the endoderm of newly produced stolons and polyps [44, 59, 63]. And in *Aurelia*, the amoebocyte, which is associated with wound healing or regeneration in non-Hydrozoa species, is transiently found in the epithelia and mesoglea during all life stages [64]. However much exploratory research using cell lineage-tracing techniques and transcriptome analyses is necessary to verify the potentiality of different cell types and the pathways regulating reversal in cnidarian life cycles, as they could probably serve as unique experimental conditions to understand how regulatory networks of gene expression and their attendant cell behaviors may control the directionality of ontogeny [65, 66].

The modifications of a life cycle are the outcome of evolution of life cycle stages, population dynamics, and adaptation to the changing environments [67–70]. Considering that neither *T*. *dohrnii* nor *L*. *undulata* are dominant species, the ecological advantage of reverse development in Hydrozoan species are not obvious [45], but the discovery of life cycle reversal in *Aurelia* may provide some critical benefits to research on jellyfish ecology. And the life cycle reversal potential of medusae or their fragments, together with their regenerative capability, should also lead to re-evaluation of the various countermeasures against blooming jellyfishes, such as the autonomous jellyfish removal robot system deployed in South Korea that ‘grinds them into a pulp that disperses in the water’ [71]. However, the success rate and duration of direct polyp formation from *Aurelia* medusae largely varied with experimental conditions, and these unique cases are only observed in the lab by far. Thus until comprehensive studies concerning to what extent it spreads among species and how frequently it occurs in the field are accomplished, the ecological significance of life cycle reversal in Cnidaria remains to be explored.

## Supporting Information

S1 Table COI and ITS sequences of *Aurelia* analyzed in this study. (DOCX)
