# Supplementary material for: Life Cycle Reversal in Aurelia sp.1 (Cnidaria, Scyphozoa)
Source: PLoS One. 2015 Dec 21;10(12):e0145314. doi: 10.1371/journal.pone.0145314 (PMC4687044; doi:10.1371/journal.pone.0145314)
Supplement: S1 Table — (DOCX) [file pone.0145314.s001.docx]

S1 Table. *Aurelia* spp. COI and ITS sequences analyzed in this study.

| COI | | ITS | |
| --- | --- | --- | --- |
| Accession | Species | Accession | Species |
| AY903117 | Aurelia aurita clone Bosporus2 | AY319848 | Aurelia aurita strain Bjornsund |
| AY903118 | Aurelia aurita clone Gullmar15 | AY319849 | Aurelia aurita strain Charlestown |
| AY903093 | Aurelia aurita clone BostonHbr1 | FR851959 | Aurelia aurita 7_1E |
| AY903094 | Aurelia aurita clone BostonHbr2 | FR851960 | Aurelia aurita 7_3E |
| AY903095 | Aurelia aurita clone BostonHbr3 | FR851961 | Aurelia aurita bBosphorus |
| AY903208 | Aurelia aurita clone Anglesey01 | FR851962 | Aurelia aurita cBosphorus |
| AY903209 | Aurelia aurita clone Anglesey02 | FR851963 | Aurelia aurita 1WhiteSea |
| AY903210 | Aurelia aurita clone Anglesey03 | FR851964 | Aurelia aurita 6WhiteSea |
| AY903211 | Aurelia aurita clone Anglesey04 | FR851965 | Aurelia aurita 16Gullmar |
| AY903212 | Aurelia aurita clone Anglesey05 | FR851966 | Aurelia aurita 17Gullmar |
| AY903068 | Aurelia labiata clone Sooke1 | FR851967 | Aurelia aurita 30Greenwich |
| AY903069 | Aurelia labiata clone Sooke2 | FR851968 | Aurelia aurita 8EBlackSea |
| AY903070 | Aurelia labiata clone Sooke3 | FR851969 | Aurelia aurita dBosphorus1 |
| AY903071 | Aurelia labiata clone Sooke5 | FR851970 | Aurelia aurita 5WhiteSea |
| AY903072 | Aurelia labiata clone Todd2 | AY935205 | Aurelia aurita clone RhodeIsland_Charlestown25 |
| AY903073 | Aurelia labiata clone Todd4 | AY935206 | Aurelia aurita clone Bjornsund20 |
| AY903074 | Aurelia labiata clone NewportBay3 | AY319842 | Aurelia labiata strain Monterey |
| AY903075 | Aurelia labiata clone TomalesBay2 | AY319843 | Aurelia labiata strain Sooke1 |
| AY903076 | Aurelia labiata clone TomalesBay3 | AY319844 | Aurelia labiata strain Sooke2 |
| AY903077 | Aurelia labiata clone TomalesBay4 | AY935202 | Aurelia labiata clone Alaska4 |
| AY903189 | Aurelia limbata clone A. limbata | AY935215 | Aurelia limbata clone AJ13h |
| AY903078 | Aurelia sp. 1 clone MarinaDelRey2 | AY935203 | Aurelia sp. 1 clone California_MdR4 |
| AY903079 | Aurelia sp. 1 clone MarinaDelRey3 | AY935214 | Aurelia sp. 1 clone #1 MiyazuBay |
| AY903080 | Aurelia sp. 1 clone MarinaDelRey4 | AY319845 | Aurelia sp. 1 strain SanDiego1 |
| AY903081 | Aurelia sp. 1 clone MarinaDelRey5 | AY319846 | Aurelia sp. 1 strain NewportBeach |
| AY903082 | Aurelia sp. 1 clone LongBeach_2 | AY319850 | Aurelia sp. 2 strain Brazil9 |
| AY903083 | Aurelia sp. 1 clone LongBeach_3 | AY319851 | Aurelia sp. 2 strain Brazil |
| AY903084 | Aurelia sp. 1 clone LongBeach_4 | AY935204 | Aurelia sp. 2 clone Brazil9 |
| AY903085 | Aurelia sp. 1 clone LongBeach_5 | AY319836 | Aurelia sp. 3 strain CRRF48 |
| AY903086 | Aurelia sp. 1 clone NewportBeach1 | AY319837 | Aurelia sp. 3 strain TKCU13B |
| AY903087 | Aurelia sp. 1 clone NewportBeach3 | AY935209 | Aurelia sp. 3 clone CRRF48 |
| AY903088 | Aurelia sp. 1 clone NewportBeach4 | AY319834 | Aurelia sp. 4 strain OLO30 |
| AY903089 | Aurelia sp. 1 clone SanDiego1 | AY319835 | Aurelia sp. 4 strain OLO31 |
| AY903090 | Aurelia sp. 1 clone SanDiego2 | AY935208 | Aurelia sp. 4 clone 2_OLO31 |
| AY903091 | Aurelia sp. 1 clone SanDiego3 | AY319840 | Aurelia sp. 5 strain Mljet4 |
| AY903092 | Aurelia sp. 1 clone SanDiego4 | AY319841 | Aurelia sp. 5 strain Mljet3 |
| AY903116 | Aurelia sp. 1 clone TokyoBay | AY935210 | Aurelia sp. 5 clone Croatia_Mljet4 |
| AY903127 | Aurelia sp. 1 clone Perth2 | AY319838 | Aurelia sp. 6 strain Helen3 |
| AY903128 | Aurelia sp. 1 clone Mooloolaba1 | AY319839 | Aurelia sp. 6 strain Helen5 |
| AY903130 | Aurelia sp. 1 clone Sydney_1 | AY935207 | Aurelia sp. 6 clone HelenReef#5-2 |
| AY903131 | Aurelia sp. 1 clone Sydney_2 | AY935212 | Aurelia sp. 7 clone North Adriatic2 |
| AY903142 | Aurelia sp. 1 clone Port_Hacking_3 | AY935213 | Aurelia sp. 7 clone BayofSton1 |
| AY903143 | Aurelia sp. 1 clone Darling_Harbour_1 | AY935217 | Aurelia sp. 8 clone MDJ17_AH3 |
| AY903146 | Aurelia sp. 1 clone Coila_Lake_1 | AY935218 | Aurelia sp. 8 clone MDJ20_ANZ2 |
| AY903147 | Aurelia sp. 1 clone Coila_Lake_3 | AY935216 | Aurelia sp. 9 clone MDJ12_AM2a |
| AY903148 | Aurelia sp. 1 clone Coila_Lake_4 | AY319847 | Aurelia sp. 10 strain Kachemack |
| AY903149 | Aurelia sp. 1 clone Coila_Lake_5 | AY935211 | Aurelia sp. 10 clone limbata_Alaska |
| AY903150 | Aurelia sp. 1 clone Coila_Lake_6 | KF962383 | Aurelia sp. 1 JRH-2014 voucher XMAS1 |
| AY903151 | Aurelia sp. 1 clone Huon_Estuary_5 | KF962384 | Aurelia sp. 1 JRH-2014 voucher XMAS2 |
| AY903152 | Aurelia sp. 1 clone Lake_Illawarra_1 | KF962385 | Aurelia sp. 1 JRH-2014 voucher XMAS3 |
| AY903153 | Aurelia sp. 1 clone Lake_Illawarra_5 | KF962386 | Aurelia sp. 1 JRH-2014 voucher XMAS4 |
| AY903154 | Aurelia sp. 1 clone Lake_Illawarra_6 | KF962387 | Aurelia sp. 1 JRH-2014 voucher XMAS5 |
| AY903155 | Aurelia sp. 1 clone Tuggerah_Lake_1 | KF962388 | Aurelia sp. 1 JRH-2014 voucher XMAS6 |
| AY903156 | Aurelia sp. 1 clone Tuggerah_Lake_2 |  |  |
| AY903157 | Aurelia sp. 1 clone Tuggerah_Lake_3 |  |  |
| AY903158 | Aurelia sp. 1 clone Tuggerah_Lake_4 |  |  |
| AY903159 | Aurelia sp. 1 clone Tuggerah_Lake_5 |  |  |
| AY903160 | Aurelia sp. 1 clone Tuggerah_Lake_6 |  |  |
| AY903161 | Aurelia sp. 1 clone Lake_Macquarie_1 | |  |
| AY903162 | Aurelia sp. 1 clone Lake_Macquarie_2 | |  |
| AY903163 | Aurelia sp. 1 clone Lake_Macquarie_3 | |  |
| AY903164 | Aurelia sp. 1 clone Lake_Macquarie_4 | |  |
| AY903165 | Aurelia sp. 1 clone Lake_Macquarie_5 | |  |
| AY903166 | Aurelia sp. 1 clone Lake_Macquarie_6 | |  |
| AY903167 | Aurelia sp. 1 clone Mooloolaba_3 |  |  |
| AY903168 | Aurelia sp. 1 clone Miyazu1 |  |  |
| AY903169 | Aurelia sp. 1 clone Miyazu2 |  |  |
| AY903170 | Aurelia sp. 1 clone Miyazu3 |  |  |
| AY903171 | Aurelia sp. 1 clone Miyazu5 |  |  |
| AY903177 | Aurelia sp. 1 clone Perth_3 |  |  |
| AY903178 | Aurelia sp. 1 clone Perth_5 |  |  |
| AY903179 | Aurelia sp. 1 clone Perth_6 |  |  |
| AY903180 | Aurelia sp. 1 clone Perth_7 |  |  |
| AY903181 | Aurelia sp. 1 clone Sydney_3 |  |  |
| AY903182 | Aurelia sp. 1 clone Sydney_4 |  |  |
| AY903183 | Aurelia sp. 1 clone Sydney_5 |  |  |
| AY903184 | Aurelia sp. 1 clone Sydney_6 |  |  |
| AY903185 | Aurelia sp. 1 clone Newport_Beach_5 | |  |
| AY903186 | Aurelia sp. 1 clone Sakata Bay 1 |  |  |
| AY903187 | Aurelia sp. 1 clone Sakata Bay 3 |  |  |
| AY903188 | Aurelia sp. 1 clone Sakata Bay 4 |  |  |
| AY903190 | Aurelia sp. 1 clone Uwa1 |  |  |
| AY903191 | Aurelia sp. 1 clone Uwa4 |  |  |
| AY903192 | Aurelia sp. 1 clone Uwa6 |  |  |
| AY903193 | Aurelia sp. 1 clone Uwa7 |  |  |
| AY903194 | Aurelia sp. 1 clone Ondo1 |  |  |
| AY903195 | Aurelia sp. 1 clone Ondo2 |  |  |
| AY903196 | Aurelia sp. 1 clone Ondo3 |  |  |
| AY903197 | Aurelia sp. 1 clone Ondo4 |  |  |
| AY903198 | Aurelia sp. 1 clone Ondo5 |  |  |
| AY903199 | Aurelia sp. 1 clone Ondo6 |  |  |
| AY903200 | Aurelia sp. 1 clone Ondo7 |  |  |
| AY903201 | Aurelia sp. 1 clone Ondo8 |  |  |
| AY903202 | Aurelia sp. 1 clone Ondo9 |  |  |
| AY903203 | Aurelia sp. 1 clone TokyoBay01 |  |  |
| AY903204 | Aurelia sp. 1 clone TokyoBay02 |  |  |
| AY903205 | Aurelia sp. 1 clone TokyoBay03 |  |  |
| AY903206 | Aurelia sp. 1 clone TokyoBay04 |  |  |
| AY903207 | Aurelia sp. 1 clone TokyoBay07 |  |  |
| AY903126 | Aurelia sp. 1 clone Perth1a |  |  |
| AY903119 | Aurelia sp. 2 clone Morandini9 |  |  |
| AY903120 | Aurelia sp. 2 clone Morandini10 |  |  |
| AY903121 | Aurelia sp. 2 clone Morandini11 |  |  |
| AY903122 | Aurelia sp. 2 clone Morandini12 |  |  |
| AY903096 | Aurelia sp. 3 clone Malakal48 |  |  |
| AY903112 | Aurelia sp. 3 clone TKCU12 |  |  |
| AY903113 | Aurelia sp. 3 clone TKCU13 |  |  |
| AY903114 | Aurelia sp. 3 clone TketauLM36 |  |  |
| AY903115 | Aurelia sp. 3 clone 41Risong |  |  |
| AY903097 | Aurelia sp. 4 clone HLM7 |  |  |
| AY903098 | Aurelia sp. 4 clone HLM26 |  |  |
| AY903101 | Aurelia sp. 4 clone OTM4 |  |  |
| AY903102 | Aurelia sp. 4 clone OTM2 |  |  |
| AY903107 | Aurelia sp. 4 clone OngaelLO29 |  |  |
| AY903108 | Aurelia sp. 4 clone OngaelLO28 |  |  |
| AY903109 | Aurelia sp. 4 clone OngaelLO31 |  |  |
| AY903110 | Aurelia sp. 4 clone BJLK1 |  |  |
| AY903111 | Aurelia sp. 4 clone BJLK5 |  |  |
| AY903136 | Aurelia sp. 4 clone AlaWaiOahu1 |  |  |
| AY903137 | Aurelia sp. 4 clone AlaWaiOahu2 |  |  |
| AY903144 | Aurelia sp. 4 clone Kkbn2 |  |  |
| AY903145 | Aurelia sp. 4 clone Kkbn3 |  |  |
| AY903123 | Aurelia sp. 5 clone Benovic2 |  |  |
| AY903124 | Aurelia sp. 5 clone Benovic4 |  |  |
| AY903125 | Aurelia sp. 5 clone Benovic6 |  |  |
| AY903103 | Aurelia sp. 6 clone HelenReef1 |  |  |
| AY903104 | Aurelia sp. 6 clone HelenReef4 |  |  |
| AY903105 | Aurelia sp. 6 clone HelenReef5 |  |  |
| AY903106 | Aurelia sp. 6 clone HelenReef6 |  |  |
| AY903099 | Aurelia sp. 6 clone NgellChannel45 |  |  |
| AY903100 | Aurelia sp. 6 clone NgellChannel46 |  |  |
| AY903129 | Aurelia sp. 6 clone NewBritain2 |  |  |
| AY903138 | Aurelia sp. 7 clone Tasmania1 |  |  |
| AY903139 | Aurelia sp. 7 clone Tasmania4 |  |  |
| AY903140 | Aurelia sp. 7 clone Tasmania12 |  |  |
| AY903141 | Aurelia sp. 7 clone Tasmania15 |  |  |
| AY903132 | Aurelia sp. 8 clone NorthAdriatic1 |  |  |
| AY903133 | Aurelia sp. 8 clone NorthAdriatic2 |  |  |
| AY903134 | Aurelia sp. 8 clone BayofSton1 |  |  |
| AY903135 | Aurelia sp. 8 clone BayofSton2 |  |  |
| AY903172 | Aurelia sp. 9 clone Gulf_of_Mexico_A_1 | |  |
| AY903173 | Aurelia sp. 9 clone Gulf_of_Mexico_A_2 | |  |
| AY903174 | Aurelia sp. 9 clone Gulf_of_Mexico_B_3 | |  |
| AY903175 | Aurelia sp. 9 clone Gulf_of_Mexico_B_4 | |  |
| AY903176 | Aurelia sp. 9 clone Gulf_of_Mexico_C_5 | |  |
| AY903067 | Aurelia sp. 10 clone limbata |  |  |
| AY903213 | Aurelia sp. 11 clone Kwajalein1 |  |  |
| KF962060 | Aurelia sp. 1 JRH-2014 voucher XMAS1 | |  |
| KF962061 | Aurelia sp. 1 JRH-2014 voucher XMAS2 | |  |
| KF962062 | Aurelia sp. 1 JRH-2014 voucher XMAS3 | |  |
| KF962063 | Aurelia sp. 1 JRH-2014 voucher XMAS4 | |  |
| KF962064 | Aurelia sp. 1 JRH-2014 voucher XMAS5 | |  |
| KF962065 | Aurelia sp. 1 JRH-2014 voucher XMAS6 | |  |
| KJ733900 | Aurelia sp. 1 JRH-2014 voucher XMAuS1 | |  |
| KJ733901 | Aurelia sp. 1 JRH-2014 voucher XMAuS2 | |  |
| KJ733902 | Aurelia sp. 1 JRH-2014 voucher XMAuS3 | |  |
